# Operationalization and measurement of compulsivity across video gaming and gambling behavioral domains

**DOI:** 10.1186/s40359-023-01439-1

**Published:** 2023-11-21

**Authors:** Ismael Muela, Juan F. Navas, Juan R. Barrada, José López-Guerrero, Francisco J. Rivero, Damien Brevers, José C. Perales

**Affiliations:** 1 Department of Experimental Psychology; Mind, Brain and Behavior Research Center (CIMCYC), University of Granada, 18071, Campus de Cartuja s/n, Granada, Spain; 2 Department of Clinical Psychology, Complutense University of Madrid, Madrid, Spain; 3 Department of Psychology and Sociology, Faculty of Education, Universidad de Zaragoza, Zaragoza, Spain; 4 Louvain Experimental Psychopathology Research Group (LEP), Psychological Sciences Research Institute (IPSY), UCLouvain, Louvain-La-Neuve, Belgium

**Keywords:** Behavioral addiction, Gambling, Video gaming, Compulsivity, Validation, Measurement, Scale, Self-report

## Abstract

**Background:**

Compulsivity is the hallmark of addiction progression and, as a construct, has played an important role in unveiling the etiological pathways from learning mechanisms underlying addictive behavior to harms resulting from it. However, a sound use of the compulsivity construct in the field of behavioral addictions has been hindered to date by the lack of consensus regarding its definition and measurement. Here we capitalize on a previous systematic review and expert appraisal to develop a compulsivity scale for candidate behavioral addictions (the Granada Assessment for Cross-domain Compulsivity, GRACC).

**Methods:**

The initial scale (GRACC90) consisted of 90 items comprising previously proposed operationalizations of compulsivity, and was validated in two panel samples of individuals regularly engaging in gambling and video gaming, using exploratory structural equation modeling (ESEM) and convergence analyses.

**Results:**

The GRACC90 scale is unidimensional and structurally invariant across samples, and predicted severity of symptoms, lower quality of life, and negative affect, to similar degrees in the two samples. Additionally, poorer quality of life and negative affect were comparably predicted by compulsivity and by severity of symptoms. A shorter version of the scale (GRACC18) is proposed, based on selecting the 18 items with highest factor loadings.

**Conclusions:**

Results support the proposal that core symptoms of behavioral addictions strongly overlap with compulsivity, and peripheral symptoms are not essential for their conceptualization. Further research should clarify the etiology of compulsive behavior, and whether pathways to compulsivity in behavioral addictions could be common or different across domains.

## Background

Compulsivity refers to the experience of feeling forced or compelled to act despite awareness of serious negative consequences, and to the behavior accompanying that experience (for reviews, see [1, 2]). At a mechanistic level, compulsivity has been proposed to imply that: (a) the behavior has become goal-detached, and thus mostly automatic and inflexible (i.e., outcome expectancy valuation no longer plays a role in motivating it, as shown by insensitivity to contingency manipulation and outcome devaluation procedures [3, 4]), or (b) the individual perseveres in behaviors driven by strong short-term motives (e.g., relief of craving/withdrawal symptoms or other intense affective states [5]) despite knowing such behaviors are pernicious in the long run. Therefore, compulsivity may encompass both stimulus-driven and goal-directed control. In the words of Heather [6], “the truth about addiction lies somewhere between the extremes of free choice and no choice” (p. 31), with different etiological models differing in their relative position between these extremes (for discussions, see [7–9]).

In spite of their differences, most models converge on conceptualizing compulsivity as the hallmark of addiction progression and maintenance [1, 10]. This view is supported by translational research showing that compulsive drug use corresponds to an extreme stage of otherwise functional learning and neuroadaptation processes [11, 12]. The endpoint of this process could be either the formation of inflexible habits, or the abnormal valuation of addictive (relative to alternative) rewards. In any case, a precise and data-driven behavioral operationalization of compulsivity should provide, first, a gateway to understanding the etiological mechanisms underlying loss of control in addictive processes. And second, it should allow researchers to identify differences and similarities between addictive disorders and other patterns of behavioral over-engagement.

A variety of non-substance-related activities, such as video gaming or Internet use, are frequently described as potentially addictive (not without controversy [13–15]). Beyond semantic arguments, operationalization and measurement of compulsivity is regarded here as a necessary step to determine its role in these candidate addictions, in comparison to well-established ones, as gambling disorder or substance use disorders (SUDs). This ‘intensional’ (i.e., etiology-, or process-based) approach [16] differs from the ‘extensional’ one adopted by the dominant components model of behavioral addiction [17], according to which an addictive behavioral pattern is defined by the co-occurrence of a set of criteria (salience, withdrawal, tolerance, relapse, mood modification, and conflict). On the one hand, the components model does not distinguish between behaviors and ensuing harms [18], and conflates core and peripheral features of addiction [19]. On the other, flexibility and overinclusiveness in the delimitation of components allow a rather liberal application across behavioral domains (see, for example [20, 21]). This has caused a proliferation of new candidate addictions and tools to measure them, and an elevated risk of overdiagnosis and unnecessary psychiatrization of everyday life [22, 23]. Along these lines, some authors have criticized the application of the addiction framework to understand conditions such as problematic Internet use (PIU) or (Internet) gaming disorder (IGD), and propose instead that these conditions are better conceptualized as resulting from their use to cope with life problems or compensate for lack of life skills [24].

In that context, the overarching aim in the present study is to advance in defining and measuring compulsivity clearly enough to gauge its presence in different domains of potentially addictive behavior. To this date, attempts in this direction have been hindered by the current state of conceptual vagueness regarding compulsivity. With that goal in mind, here we capitalize on a recent systematic review by Muela et al. [25], who carefully analyzed available measures of behavioral addiction in search for items that could be considered sensitive to compulsivity. Bottom-up item content analysis and synthesis yielded six different possible operationalizations of compulsivity (see Table 1). Importantly, these operationalizations largely overlapped with the ones identified using more theoretically driven, top-down, approaches [10, 26].

Muela et al. [25] also used an expert appraisal procedure to detect delimitation problems in items included in these operationalizations of compulsivity, with the most important problems being that (a) many items mentioned negative consequences but not disutility (i.e., net imbalance between harm and reward); and (b) many items that mentioned feeling compelled towards the problematic activity made no mention of loss of control, or inability to stop the habit or to resist the urge to engage in activity-related behavior. In other words, the most repeated comments by the experts were that being aware of negative consequences but not of the global irrationality of one’s actions (i.e., harms overcome rewards), or just experiencing a strong desire but not feeling such a desire seriously jeopardizes control, are insufficient for an item to reflect true compulsivity. As Muela et al.’s initial search just included the items as worded in the scales reviewed, many of the items lacked the specificity required to pin compulsive behaviors down. In consequence, in the present work, further steps were taken to develop a sufficiently valid, sensitive, and discriminative measure of compulsivity that can be applied across behavioral domains.

**Table 1:** Operationalizations of compulsivity. (adapted from Muela et al., 2022)

Operationalization
1. Cognitive/attentional hijacking or interference caused by activity-related thoughts or images
2. Insuperable urge compelling the individual towards the activity that jeopardizes the ability to control it
3. Behavior continuance despite awareness of imbalance between harm and reward
4. Inability to interrupt the problematic behavior once initiated, resulting in sessions lasting longer than planned (binging)
5. Automatic behavior triggered by cues in absence of declarative instrumental goals (habit)
6. Inflexible or stereotyped behaviors or rituals regarding completion or execution of parts of the activity

Muela et al.’s review [25] resulted in the selection of 90 items representing the six proposed manifestations of compulsivity in the field of behavioral addictions. So, following the recommendations formulated in that review, in the present work part of those 90 items were reworded to explicitly mention disutility or lack of control. The definitive set was administered to a convenience sample of individuals with high degrees of engagement in gambling or video gaming activities (including participants below and above clinical significance thresholds). The aims were: (a) To unveil the factor structure of the 90-items pool of compulsivity-sensitive items; (b) to examine whether compulsivity can be measured across different behavioral domains using a single instrument, i.e., to assess cross-domain structural invariance of the scale; (c) to explore differential relationships of compulsivity (or its components) with correlates of gaming/gambling problems, i.e., negative affect, quality of life, and gaming/gambling motives; and, (d) to order the 90 items based on of their ability to capture any factor/s previously identified. That ordering should result in a shortened and more usable version of the questionnaire, to be employed in further research.

Although the six operationalizations of compulsivity are conceptually separable, our hypotheses remained open regarding the factorial composition of the scale. That is, we do not necessarily expect such operationalizations to correspond to separable dimensions of compulsivity. Hence, exploratory factor analyses were used to assess the structure of the compulsivity scale.

Our predictions were more specific with regard to differential correlations across gambling and gaming domains. Previous theoretical reviews [9, 27] have proposed that gambling problems are crucially driven by structural features of gambling devices that interfere with the normal functioning of the reinforcement learning system, similar to how addictive drugs do. Video gaming-related problems were conceptualized in those reviews as resulting from overvaluation of gaming and game-related outcomes, rather than mostly automatic conditioning processes. We thus hypothesize gambling problems to be more driven by compulsivity than video gaming-related problems. Hence, we expect to find stronger negative correlations of compulsivity with quality-of-life, negative affect, and severity scores in the gambling sample than in the video gaming one, under the assumption that wellbeing reduction in problem video gamers would more substantially accounted by factors other than compulsivity.

## Methods

### Participants and procedure

Participants were members of a Spanish online panel (following the UNE ISO 20252 and ESOMAR standards). The online survey was offered to active panel members being at least 18 years old. Potential candidates received an invitation via email to participate in the study. Before their potential inclusion, candidates were told this was a study for academic purposes, were warranted confidentiality and anonymity, and were informed about the estimated duration of the survey, and incentive conditions. The panel provider uses a financial compensation system based on points that can be redeemed through different online payment partners or be paid directly into panelist´s bank accounts.

Two pools of potential participants playing video games or gambling games were contacted. The main inclusion criterion for each of them was self-categorizing as being a regular gambler or a regular video gamer. Lottery-only gamblers were not eligible for the study, and the same participant was not eligible for the two samples. After acceptance, participants were provided detailed information about the specific procedure and general aims of the study, and were informed they could abandon the study at any time. After explicit informed consent, access to the full behavioral survey was granted, starting with the initial ad-hoc questionnaire to collect sociodemographic and gambling or gaming participation information.

The following measures were taken to ensure data quality. The response to the question on the preferred gambling or gaming modality in the initial survey was contrasted with responses to the last part of the full behavioral survey, including yes/no questions for having used each game type. If there was no correspondence between these answers (e.g., reporting First Person Shooter as their favorite game genre and then answering not having played that type of game) the participant was discarded. Additionally, two control items were included in the full survey. In the first one, the participant was simply asked to select a specific response option. In the second one, the participant was asked to select the lowest number in a series. If the response in any of these items was not the one instructed, the participant was discarded.

Recruitment continued until reaching a minimum of 300 fully valid and complete surveys in each subsample, which finally yielded 312 participants in the gambling sample and 319 in the gaming sample with no missing data. 39.74% of the participants who gamble presented a score equal to or above the cut-off for gambling disorder (4 or more symptoms present, according to the GD9 instrument described below), whereas the percentage of participants who played video games with scores equal to or above the 5-symptom cut-off for clinical significance was 29.68% (according to the IGD instrument described below).

### Measures

The instruments used in the entire protocol for both the video gaming and the gambling samples are available (in Spanish) in the accompanying Open Science Framework (OSF) link (see Availability of data and materials section).

#### Initial ad-hoc survey

The initial questionnaire collected quantitative information on age, and categorical information on education level, monthly income, and the modality of video game or gambling games they preferred. In the case of video gaming the options were (i) Multiplayer Online Battle Arena (MOBA); (ii) Massively Multiplayer Online Role-playing Game (MMORPG); (iii) Battle Royale; (iv) First Person Shooters (FPS); (v) strategy games, (vi) fighting or sport games; (vii) action games; (viii) mobile games; and (ix) other games not listed above. In the case of gambling the list included (i) scratchcards, (ii) card games, (iii) bingo, (iv) slot machines, (v) roulette, (vi) sport bets, and (vii) other games not listed above. In both lists, there was a last option for ‘none’.

#### 90-item Granada assessment for cross-domain compulsivity (GRACC90)

As noted earlier, this questionnaire is the target measure in the present study, and was developed after Muela et al.’s [25] selection of compulsivity-sensitive items. In their systematic review, these items were assessed by a pool of experts, who identified potential delimitation problems in the items from some categories. In order to solve such delimitation problems “negative consequences” items were reworded to explicitly mention the irrationality of behaviour, or perceiving negative consequences as costlier than potential benefits (e.g., “I cannot quit playing, despite it is causing me more harm than good”); and “urge/craving” items were reworded when necessary to explicitly mention lack of control (e.g., “I can’t control the urge to start playing”). All items were worded and administered in Spanish. Gambling and gaming versions of all items were identical except for the target activity (video gaming, gambling). Responses were collected using a 5-point scale (from 1 = *totally disagree* to 5 = *totally agree*). The complete pool of items and their English translation can be found in the accompanying Open Science Framework (OSF) link for Supplementary Materials, Data, and Code (see Availability of data and materials section). Note, however, that the English version has not been validated and is reported here for information purposes only.

Despite this attempt to better delineate the items, and to make them as discriminative as possible, we are aware that items cannot be completely free of delimitation problems. Indeed, some degree of over-inclusiveness, in combination with the large number of items, is recommendable at this stage [28], in order to further assess item quality based on participants’ responses.

#### Quality of life in individuals addicted to psychoactive substances test (TECVASP)

This instrument [29] consists of 22 5-point Likert-type items, ranging from 1 = *a lot* to 5 = *not at all*, assessing perception of physical and psychological wellbeing and health, both in general and in relation to substance use. For the purposes of this study, this questionnaire was slightly adapted by rewording items mentioning drug use as referring to video gaming or gambling. A higher score represents better quality of life. Internal consistency, as measured by Cronbach’s alpha, was 0.86. (All reported Cronbach’s alphas –for this scale and all the following ones– correspond to the samples of the present study).

#### Diagnostic questionnaire for gambling disorder (GD9)

This measure [30] (Spanish validation, [31]) was originally used to measure severity of gambling-related problems, and consists of 19 items that evaluate the ten diagnostic criteria for pathological gambling in the DSM-IV-TR [32]. Since the illegal acts criterion was eliminated in the DSM-5 diagnosis for gambling disorder, the answers to the 17 items that explore the 9 DSM-5 symptoms of gambling disorder have been used for the present study (α = 0.89). The present DSM-5-adapted version has also been satisfactorily validated in previous studies [33].

#### Diagnostic questionnaire for internet gaming disorder (IGD9)

This scale was used to measure severity of video gaming-related problems. The nine IGD criteria proposed in Section III (emerging conditions) of the DSM-5 [34] were assessed with 9 items (one per criteria) as proposed by Mallorquí-Bagué et al. [35]. The cut-off point for the diagnosis of IGD is set at 5 or more criteria. This particular form of the questionnaire was used instead of the more common IGDS9-BF (assessing the same criteria with Likert scales [36]) to allow the highest possible degree of comparability between this and the GD9 measure, without any loss of reliability (α = 0.85 in the current sample).

#### Negative affect scale of the positive and negative affect scales (PANAS)

Only the negative affect subscale of the PANAS [37] (Spanish version [38]) was used here, i.e., a general dimension of psychological distress composed of 10 adjectives (e.g., “Nervous”). Responses were collected using a 4-point Likert scale representing how well the adjective describes how the participant has felt in the last week (0 = *nothing*, 3 = *a lot*; α = 0.92).

#### Brief gambling motives Inventory (bGMI)

The bGMI [39] assesses four gambling motives with 18 items with response options ranging from 0 = *never/almost never* to 3 = *always/almost always*. The four dimensions are Affect Regulation (7 items; e.g., “To forget my worries”; α = 0.94), Financial (4 items; e.g., “To win money”; α = 0.85), Fun/Thrill (4 items; e.g., “Because it’s fun”; α = 0.83), and Social motives (3 items; e.g., “Because it makes a social gathering more enjoyable”; α = 0.72).

#### Video gaming motives questionnaire (VMQ)

The VMQ [40] assesses eight video gaming motives with 24 items (three for each dimension) with response options ranging from 1 = *strongly disagree* to 5 = *strongly agree*. The eight dimensions are Cognitive Development (e.g., “Games make me think”; α = 0.68), Competition (e.g., “I like to win”; α = 0.76), Coping (e.g., “It helps me get rid of stress”; α = 0.82), Customization (e.g., “I enjoy customizing things in games”; α = 0.81), Fantasy (e.g., “I enjoy putting myself into a new character’s shoes in each game”; α = 0.87), Recreation (e.g., “I enjoy gaming”; α = 0.83), Social Interaction (e.g., “I make new friends”; α = 0.88), and Violent Reward (e.g., “I like violence in video games, the more violent the better”; α = 0.89).

#### Final ad-hoc survey

A final survey was used to evaluate the self-assessed degree of involvement in gaming or gambling activities, classifying them into the same modalities referred to in the initial ad-hoc survey. For each gaming/gambling modality, participants were asked first to report whether they had played a specific modality in the past 12 months. If the answer was affirmative, they were asked to answer two additional questions on frequency and money spent in a typical day, in the case of gambling, and time (instead of money), in the case of gaming. The survey finished with two general questions on weekly time spent and monthly monetary expenditure for the totality of gaming/gambling activities. Only these two final questions were used for analysis in the present study. For video games, participants were told that monetary expenditure referred to any kind of game-related transaction or purchase, including the game itself, supplementary software, upgrades, in-game microtransactions, and gaming gear. For gambling, monetary expenditure referred to net loss.

### Statistical analyses

We analyzed the internal structure of GRACC90 scores with an exploratory structural equation model (ESEM [41]). Although item generation was completed according to a multicomponential theoretical model, the number of factors to be retained could not be anticipated before data analysis. Thus, the number of factors was determined by parallel analysis [42], visual inspection of the scree-plot, theoretical interpretability of the solutions, factor simplicity, and loading sizes. This analysis was done first for the full (combined) sample and, after deciding the number of factors, we tested the resulting model with the gambling and video gaming samples separately.

Subsequently, a factor invariance analysis according to type of activity (gambling or video gaming) was also carried out. In order to test invariance, the equality (or minimal difference) of the fit between consecutive models was evaluated. First, we tested the equality of form. In ESEM, this involves fixing the number of factors and pairs of correlated uniqueness (if any). Subsequently, we tested the equality of thresholds and factor loadings across groups. We considered these restrictions to be satisfactorily met if the decrease in CFI was lower than 0.01 and RMSEA increased by less than 0.015 [43, 44]. Models were analyzed using robust weighted least squares (WLSMV estimator in MPlus). According to conventional cut-offs [45], values greater than 0.95 for the comparative fit index (CFI) and Tucker-Lewis index (TLI) are considered to be indicative of an adequate and excellent fit to the data, respectively, whereas values smaller than 0.06 for the root mean square error of approximation (RMSEA) and smaller than 0.08 for standardized root mean square residual (SMSR) are indicative of acceptable model fit. It should be noted that these cut-offs were developed for confirmatory factor analysis with continuous responses, so these values should be interpreted with caution [46]. Additionally, these cut-off values should be considered as rough guidelines and not interpreted as “golden rules” [47].

We developed a short version of the instrument by selecting the items with highest loadings and inspection of their content. With this brief version (GRACC18), we repeated the same analysis to test its internal structure.

Internal consistencies of the different scales scores were then computed with Cronbach’s alpha. We computed Pearson correlations between the different dimensions of the GRACC90 scores and the other measures splitting by type of activity.

The analyses were performed with Mplus 8.4 [48] and R 4.2.2 [49]. The open database and code files for these analyses are available at the analysis folder of the OSF site for supplementary materials (see Availability of data and materials section).

## Results

### Descriptive analyses

Sociodemographic data for the two samples, as well as measure of involvement in the main activity of interest are shown in Table 2.

**Table 2:** Descriptive statistics for the participants in the two samples

	Gamblers	Gamers
	n = 312	n = 319
**Educational Level**	Number (percentage)	
No formal studies/Compulsory education not finished	7 (2.3%)	5 (1.5%)
Compulsory education finished	26 (8.3%)	22 (6.9%)
High school/Professional training not finished	58 (18.6%)	43 (13.5%)
High school/Professional training finished	92 (29.5%)	93 (29.2%)
University studies not finished	35 (11.2%)	27 (8.5%)
University studies finished	94 (30.1%)	129 (40.4%)
**Household monthly income**		
Less than 1000 euros	31 (10.0%)	29 (9.1%)
Between 1001 and 1500 euros	45 (14.4%)	56 (17.6%)
Between 1501 and 2000 euros	72 (23.1%)	63 (19.7%)
Between 2001 and 2500 euros	74 (23.7%)	88 (27.6%)
More than 2500 euros	90 (28.8%)	83 (26.0%)
**Gender**		
Male	137 (43.9%)	147 (46.1%)
Female	172 (55.1%)	171 (53.6%)
Other	3 (1%)	1 (0.3%)
	Mean (Standard deviation)	
**Age (years)**	37.9 (11.8)	40.4 (10.9)
	Median [25-75th percentile]	
**Weekly time spent (hours)**	3.0 [1.0-9.25]	9.0 [4.0-17.5]
**Monthly expenditure (euros)**	31.0 [10.0-135.0]	7.0 [0.0–20.0]

Notes: Gambling severity was assessed with GD9 and gaming severity, with IGD9. For weekly time and monthly money invested, the median and lower and upper bounds of the interquartile range were calculated instead of mean and standard deviation to avoid the influence of extreme scores

The two samples were well matched on all demographic characteristics, but differed – as expected – in expenditure of time and money, with gamers spending longer hours in their main activity of interest, and gamblers larger sums of money.

### Internal structure and consistency of the GRACC90

**Fig. 1:**
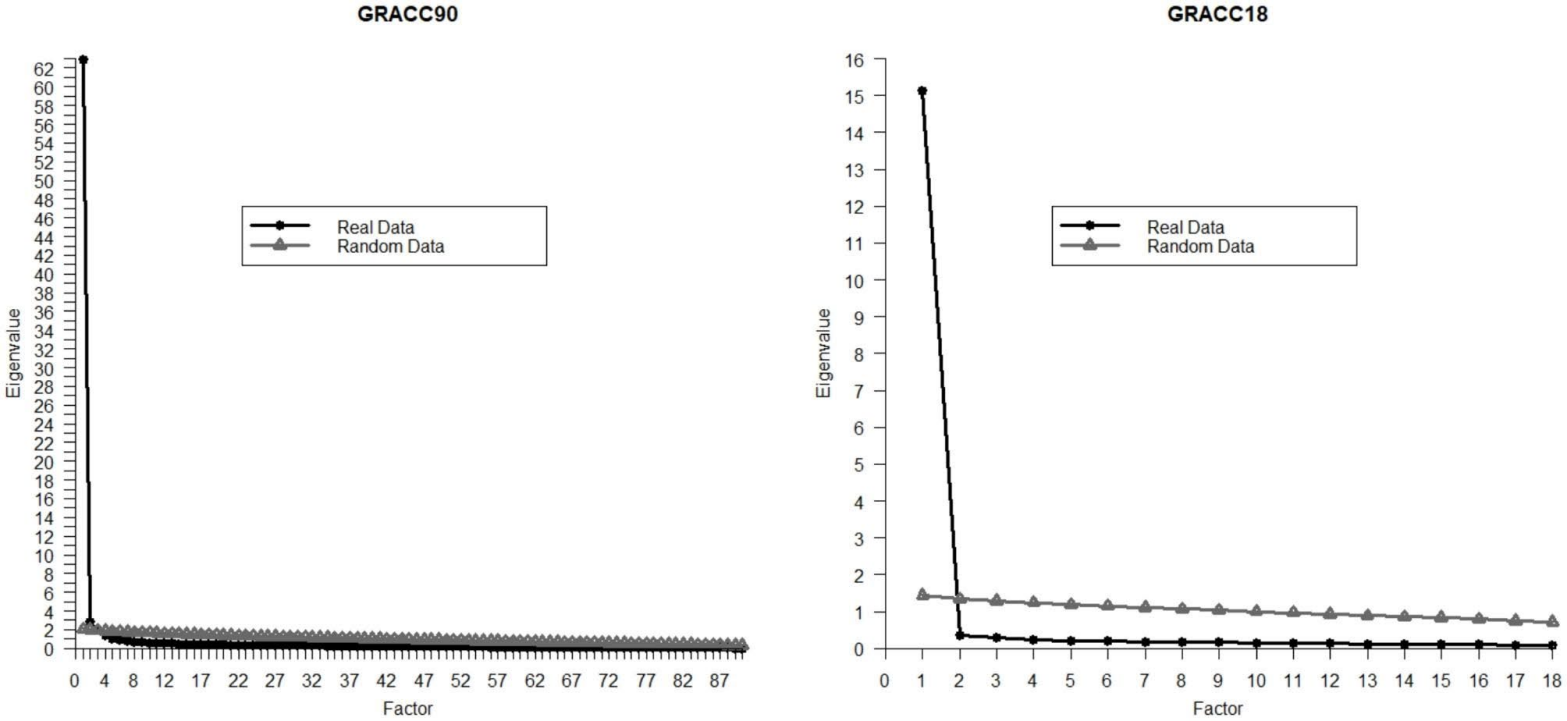
Scree-plot and results of the parallel analysis for the full (90-item; GRACC90) and short (18-item; GRACC18) versions of the compulsivity questionnaire

The scree-plot and the results of the parallel analysis are displayed in Fig. 1. In the scree-plot, a single eigenvalue clearly outstood relative to the others. In the parallel analysis, three eigenvalues from the sample (62.85, 2.76, and 2.04) were greater than the eigenvalues from the randomly generated datasets (2.07, 1.98, and 1.92). In view of this, we tested uni-, bi-, and three-dimensional solutions.

Fits of the different models are shown in Table 3. The unidimensional solution was satisfactory (CFI = 0.971, TLI = 0.970, RMSEA = 0.054, SRMR = 0.039). Although model fit was slightly improved in a bidimensional model (CFI = 0.980, TLI = 0.979, RMSEA = 0.045, SRMR = 0.029), that second factor could not be theoretically interpreted. Only three items presented higher loadings in that second factor than in the first one (loadings in the range [0.50, 0.59]), but, even in those cases, relevant cross-loadings to the first factor were present (loadings in the range [0.26, 0.47]). Again, fit was improved in the three-factor model (CFI = 0.987, TLI = 0.986, RMSEA = 0.037, SRMR = 0.023), but no items showed a higher loading in the third dimension than in the first one.

**Table 3:** Goodness-of-fit indices for the different models

	*χ* ^2^	*df*	CFI	TLI	RMSEA	SRMR	ΔCFI	ΔRMSEA
**Full version (GRACC90)**								
Full sample								
M1. 1 factor	11,112.9	3915	0.971	0.970	0.054	0.039		
M2. 2 factors	8,752.9	3826	0.980	0.979	0.045	0.029		
M3. 3 factors	7,019.7	3738	0.987	0.986	0.037	0.023		
Subsamples								
M4. Gambling sample	6,309.6	3915	0.984	0.984	0.044	0.033		
M5. video games sample	7,504.3	3915	0.968	0.967	0.054	0.052		
*Invariance by type of activity*								
M6. Equal form	13,904.2	7830	0.977	0.976	0.050	0.044		
M7. Equal loadings and thresholds	14,325.2	8188	0.977	0.977	0.049	0.044	0.000	–0.001
**Short version (GRACC18)**								
Full sample								
M8. 1 factor	501.0	135	0.996	0.996	0.066	0.013		
Subsamples								
M9. Gambling sample	298.0	135	0.997	0.996	0.062	0.014		
M10. video games sample	407.6	135	0.994	0.993	0.080	0.019		
*Invariance by type of activity*								
M11. Equal form	713.6	270	0.995	0.995	0.072	0.017		
M12. Equal loadings and thresholds	723.7	340	0.996	0.996	0.060	0.018	0.001	–0.012

Notes. *df* = degrees of freedom; CFI = comparative fit index; TLI = Tucker-Lewis index; RMSEA = root mean square error of approximation; SRMR = standardized root mean square residual; Δ = increment in fit index with respect to previous model. All *p*-values for the chi-square test were < 0.001

Considering this, we decided to retain a single factor, as the second and third dimensions, if extracted, were residual and not interpretable. Item loadings are shown in Table S2 (see OSF link for supplementary materials in Availability of data and materials section; item order is the same as in Table S1 and the scales as presented to participants). Although the item categories, as identified by Muela et al., were not retained in this analyses, the mean loads for the eight categories differed to some extent. In the same order used for Table 1, mean loads [range] for items in each category were: 0.89 [0.83; 0.92], 0.87 [0.79; 0.92], 0.86 [0.76; 0.93], 0.83 [0.68; 0.91], 0.78 [0.48; 0.91]. and 0.77 [0.48; 0.85].

Overall, loadings were very high in this factor (*M* = 0.83; *max* = 0.93 –“I continue to play even though I’m fully aware that I have increased the risks in certain aspects of my life so much that it’s not worth it”–; *min* = 0.48 –“Often, when I’m playing, I find that my mind has drifted”–). When we tested this model in the gambling and video gaming samples separately, fit was satisfactory for both of them (CFI = 0.984/0.968, TLI = 0.984/0.967, RMSEA = 0.044/0.054, SRMR = 0.033/0.052), although slightly better for the gambling sample. Evidence that the model was invariant with respect to type of activity was obtained by comparing fits of a model with equal form and with equal loading and thresholds. The more restrictive model led to no meaningful change in fit (𝛥CFI = 0.000, 𝛥RMSEA = –0.001).

In order to meet the last aim of the study, i.e. providing a more usable scale in conditions of time constraints, we developed a shortened version of the questionnaire. Given the previously observed unidimensional structure and invariance with respect to activity, we included the 18 items with highest loadings across samples (cross-sample load ≥ 0.90, rounded to the second decimal; henceforth, GRACC18). Table 4 shows the English translation of these items, whereas the original items as worded in Spanish are available in Table S1 in the OSF link (see Availability of data and materials section).

**Table 4:** English translation of the items included in GRACC18

Item wording
I continue to play even though I’m fully aware that I have increased the risks in certain aspects of my life so much that it’s not worth it.
I feel I can’t get thoughts about playing out of my head.
Anything related to playing immediately catches my attention and interferes with what I’m doing at that moment.
I feel an uncontrollable desire to play even right after I’m done.
The game is on my mind even when I’m not playing, and I should be thinking about something else.
I often find myself thinking when I will play again, instead of focusing on what I should be doing.
I keep playing even though I am aware that the harm it does me is greater than the benefits.
I can’t stop playing, even though playing has had a negative impact on my life that clearly outweighs its positive impact.
Every time I play, I feel like I’m on a slippery slope that I can’t get back up.
Spending a lot of time playing has become an almost involuntary habit.
Sometimes, the desire to play dominates me.
I keep playing even though I feel guilty for my irrational behavior.
Once I have started, I can’t stop playing unless something external forces me to.
I often play because I feel an irrepressible desire to play when a surge of strong emotions take over me.
Often playing is something that I want to do so badly that I feel my heart beating faster.
My thoughts continuously revolve around playing, even when I’m not playing.
I can’t stop the desire to play when I’m overpowered by certain bodily or internal sensations.
I haven’t stopped playing, even though doing so is causing me more disadvantages than advantages.
Note: The items were originally worded in Spanish. Any use of the scale with an English speaking sample would require an independent validation.

The GRACC18 consisted of items 78, 74, 80, 83, 56, 82, 57, 47, 45, 46, 60, 55, 48, 39, 79, 40, 20, and 90 from the GRACC90. For this version, the parallel analysis (Fig. 1) clearly showed the convenience of retaining a single factor. Model fit indices in the GRACC18 for the full/gambling/video gaming samples were: CFI = 0.996/0.997/0.994, TLI = 0.996/0.996/0.993, RMSEA = 0.066/0.062/0.080, RMSEA = 0.013/0.014/0.019. As expected, item loadings were very high (in the range [0.88, 0.94]).

As with the full version, we found support for invariance with respect to activity, as the model with equality of loadings and thresholds implied no relevant change in fit (𝛥CFI = 0.001, 𝛥RMSEA = –0.012). As expected, in view of the item loadings and scale lengths, internal consistency indices were very high for both the GRACC90 (α = 0.99) and the GRACC18 (α = 0.98).

### Associations with other variables

Descriptives and Pearson correlations for all measures are shown in Table 5 for the gambling sample and Table 6 for the video gaming sample. The mean scores in the GRACC90 showed no statistically significant difference by type of activity [*M*_gambling_ = 2.41, *SD*_gambling_ = 1.10, *M*_video gaming_ = 2.34, *SD*_video gaming_ = 0.96, *t*(629) = 0.860, *p* = .390, *d* = 0.07].

**Table 5:** Measure descriptives (lower panel) and correlations (upper panel) between measures for the sample of gambling participants

	*GRACC90*	*GD9*	*TECVASP*	*PANAS - NA*	*bGMI* *Affect*	*bGMI* *Financial*	*bGMI* *Fun*	*bGMI* *Social*
*GRACC90*								
*Severity (GD9)*	0.81							
*QoL (TECVASP)*	− 0.60	− 0.60						
*Neg. affect (PANAS)*	0.45	0.50	− 0.73					
*bGMI - Affect*	0.77	0.74	− 0.56	0.40				
*bGMI - Financial*	0.35	0.39	− 0.30	0.30	0.35			
*bGMI - Fun*	0.55	0.51	− 0.32	0.23	0.71	0.32		
*bGMI - Social*	0.61	0.55	− 0.42	0.26	0.72	0.31	0.62	
	***GRACC90***	***GD9***	***TECVASP***	***PANAS - NA***	***bGMI*** ***Affect***	***bGMI*** ***Financial***	***bGMI*** ***Fun***	***bGMI*** ***Social***
Mean	2.41	3.08	81.18	18.62	7.03	6.56	5.74	3.04
Standard deviation	1.10	3.05	13.56	7.29	5.90	3.56	3.14	2.38
Skewness	0.34	0.59	− 0.42	0.74	0.47	− 0.20	− 0.04	0.44
Kurtosis	-1.17	-1.06	− 0.59	− 0.32	− 0.89	-1.03	− 0.79	− 0.63

Note. All the correlations are statistically significant at *p* < .05. GRACC90 scores were computed as means of item scores. For all other measures, scores are computed as sums of item scores. See Measures section for further details of the variables

**Table 6:** Measure descriptives (lower panel) and correlations (upper panel) between measures for the sample of video gaming participants

	*GRACC90*	*IGD9*	*TECVASP*	*PANAS* *NA*	*VMQ* *Dev.*	*VMQ* *Comp.*	*VMQ* *Coping*	*VMQ* *Custom*	*VMQ* *Fantasy*	*VMQ* *Recr.*	*VMQ* *Social*	*VMQ* *Violence*
*GRACC90*												
*Severity (IGD9)*	0.75											
*QoL (TECVASP)*	− 0.57	− 0.62										
*Neg. affect - PANAS*	0.35	0.49	− 0.68									
*VMQ - Development*	0.49	0.39	− 0.25	0.21								
*VMQ - Competition*	0.54	0.48	− 0.23	0.17	0.57							
*VMQ - Coping*	0.54	0.46	− 0.42	0.38	0.65	0.48						
*VMQ - Customization*	0.41	0.36	− 0.28	0.24	0.48	0.44	0.51					
*VMQ - Fantasy*	0.51	0.44	− 0.31	0.22	0.59	0.52	0.63	0.70				
*VMQ - Recreation*	0.14	0.14	0.02	0.07	0.37	0.38	0.37	0.38	0.46			
*VMQ - Social Interaction*	0.63	0.52	− 0.34	0.19	0.51	0.61	0.53	0.46	0.52	0.20		
*VMQ - Violence*	0.59	0.43	− 0.30	0.16	0.35	0.43	0.41	0.26	0.36	0.05	0.54	
	***GRACC90***	***IGD9***	***TECVASP***	***PANAS*** ***NA***	***VMQ*** ***Dev.***	***VMQ*** ***Comp.***	***VMQ*** ***Coping***	***VMQ*** ***Custom***	***VMQ*** ***Fantasy***	***VMQ*** ***Recr.***	***VMQ*** ***Social***	***VMQ*** ***Violence***
Mean	2.34	2.17	83.57	17.21	8.18	8.27	8.37	8.33	8.36	10.52	6.45	5.37
Standard deviation	0.96	2.52	11.64	6.36	2.25	2.45	2.45	2.59	2.72	1.76	2.93	2.77
Skewness	0.42	0.95	− 0.69	0.80	− 0.27	− 0.30	− 0.40	− 0.41	− 0.50	-1.07	0.29	0.84
Kurtosis	− 0.90	− 0.21	0.17	− 0.15	− 0.53	− 0.80	− 0.68	− 0.59	− 0.70	0.64	-1.18	− 0.58

Note. All the correlations were statistically significant at *p* < .05, except underlined values. GRACC90 scores were computed as means of item scores. For all other measures, scores are computed as sums of item scores. See Measures section for further details of the variables

With respect to the constructs that were assessed in both samples, GRACC90 scores presented the highest correlation with severity scores (*r*_gambling_ = 0.81, *r*_video gaming_ = 0.75), followed by Quality-of-Life scores (*r*_gambling_ = –0.60, *r*_video gaming_ = –0.57), and, to a smaller degree, with Negative Affect (*r*_gambling_ = 0.45, *r*_video gaming_ = 0.35). All correlations were statistically significant, at *p* < .001. Of interest, although all the associations were slightly higher for the gambling sample, when comparing these correlations pair-by-pair only the difference of correlation size for severity was statistically significant. For severity, *r*_difference_ = 0.06, *z* = 2.003, *p* = .045; for Quality of Life, *r*_difference_ = 0.03, *z* = 0.556, *p* = .578; for Negative Affect, *r*_difference_ = 0.10, *z* = 1.419, *p* = .156.

For the gambling sample, GRACC90 scores were positively correlated with all gambling motives (*M*_*r*_ = 0.57), with a maximum association with Affect regulation motives (*r* = .77) and a minimum association with Financial motives (*r* = .35). For the video gaming sample, GRACC90 scores were positively correlated with all gaming motives (*M*_*r*_ = 0.48), with a maximum association with Social Interaction motives (*r* = .63) and a minimum association with Recreational motives (*r* = .14). All *p*-values were < 0.001, except for the correlation with Recreational motives, *p* = .01.

GRACC90 scores and severity scores showed similar associations with Quality of Life and Negative Affect for both samples. The only statistically significant difference in correlation size was that, for the videogaming sample, severity scores overlapped to a larger degree with Negative Affect than compulsivity, *r*_difference_ = 0.14, *z* = 3.969, *p* < .001.

GRACC90 and GRACC18 scores showed an extremely high overlap (*r* = .98). Accordingly, correlations between GRACC18 scores and the other assessed constructs mimicked those obtained with GRACC90, although slightly smaller (mean change of unsigned correlations was 0.03).

## Discussion

This study was aimed at developing a scale to measure compulsivity across two domains of potentially addictive behavior. Gambling and video gaming were selected as the best available representatives of a broadly recognized addictive activity (gambling) and a strong candidate to be recognized as such (video gaming). A pool of items comprising six proposed compulsivity operationalizations was developed following the recommendations appraised by a pool of experts, as collected and assembled by Muela et al. [25]. An initial 90-item version of the survey was administered to two samples of frequent gamblers and gamers. Item wordings were identical for both samples, except for the target activity.

Responses to the compulsivity scale were best comprised by a unidimensional model. Although two- and three-factor solutions yielded slightly better model fit indices, substantial cross-loadings and lack of content coherence rendered the one-factor solution clearly superior. In line with previous studies using panel samples for similar purposes, mean severity scores and proportions of individuals above the clinical cut-off in both samples were high, relative to samples obtained with other recruitment methods [50]. In other words, our factor analysis seems to be valid for the whole severity continuum and is not compromised by scores’ range restriction. Moreover, invariance analyses showed that this structure held across samples. As shown in the OSF link for supplementary materials, item loadings were similar across domains, with loads for the most discriminative items being almost coincident in the gambling and video gaming scale versions. In other words, results do not support the view that compulsivity (at least when assessed with a self-report measure) is multifactorial, or that different dimensions could be more clearly present in one domain or the other. Regardless of its specific definition, compulsivity seems to be unidimensional and mostly not differentiable –at least when assessed with self-reports– between both domains.

We also observed that compulsivity scores were slightly more strongly correlated with severity scores in the gambling sample (*r* = .81) than in the video gaming sample (*r* = .75). Although the difference between these correlations was significant, it must be interpreted with caution. First, in both severity scales, the contents of some items strongly overlap with the ones of compulsivity items (actually, many of the scales included in Muela’s review were behavioral addiction severity scales), so the correlations could be inflated. And second, in spite of their strong similarities, gambling and gaming disorder severity scales are different instruments, and their distributions are not parallel (e.g., the diagnostic threshold for gambling disorder is four symptoms, whereas the proposed DSM5-section III cut-off for gaming disorder is five). That was the main reason to include negative affect and quality-of-life measures for the two samples in convergent validity analyses.

As expected, compulsivity was strongly and negatively correlated with quality-of-life scores (-0.60 and − 0.57 for the gambling and gaming samples, respectively), and moderately and positively correlated with negative affect (0.45 and 0.35 for the gambling and gaming samples, respectively). Correlations of compulsivity with negative affect and quality of life scores did not significantly differ across samples. Importantly, in the gambling sample, compulsivity by itself was as good as severity at predicting quality of life and negative affect. This pattern of correlations converges with recent studies that critically appraise gambling severity indices for failing to discriminate between core features of addictive behavior (e.g., lack of control or craving) and harms derived from those behaviors (e.g., missing work opportunities or jeopardizing social relationships) [18]. The unifactorial structure of behavioral addiction severity scales is likely to be attributable to the strong correlation between causes and consequences [51], but a core of psychological features seems to play a larger role in the etiology of the myriad of manifestations or ‘components’ measured by customary addiction scales.

In the video gaming sample, on the contrary, severity was a slightly but significantly better predictor of negative affect (*r* = .49) than compulsivity (*r* = .35). This could mean that the IGD severity score captures elements that contribute to reduced wellbeing in problematic video gaming that are not accounted for by compulsivity. In other words, the contribution of factors other than compulsivity to functional deterioration is probably larger in video gaming-related problems than in gambling problems. Again, however, the difference in correlations is small, and the GD9 and IGD9 measures are not exactly equivalent, so this potential difference must be interpreted with caution.

Although all items included in the GRACC90 substantially loaded to the common factor, we selected the items with highest loadings across samples in order to provide a shortened version of the questionnaire. Among the 18 best items (load ≥ 0.90, rounded to the second decimal, in both samples), five of them referred to persevering despite knowledge of the imbalance between harm and reward (items 47, 55, 57, 78, and 90), 5 to cognitive hijacking (40, 56, 74, 80, and 82), 5 to irresistible urge jeopardizing control attempts (20, 39, 60, 79, 83), and only 2 to automaticity (45, 46), and 1 to sessions lasting longer than planned/binging (48, although this item also explicitly mentions lack of control), and none to rituals/inflexible rules. At the other end, items referring to rituals/inflexible rules, sessions lasting longer than planned (binging), and automaticity are systematically among the least discriminative ones. This selection procedure obviously made the GRACC18 more neatly unidimensional than the GRACC90 (with only one eigenvalue above the eigenvalues of randomly generated samples; see Fig. 1, right panel), but did not alter at all the capacity of the scale to predict quality of life and negative affect.

Importantly, these results converge with (a) a recent machine learning analysis showing that a subset of diagnostic criteria (withdrawal, relapse, and conflict) strongly and specifically predict dysfunctional and harmful video gaming [52]; and (b) seminal works on core components of behavioral addiction, i.e. salience, withdrawal, relapse, and conflict [53]. The effect of craving/urge on diminished control is not included as a diagnostic criterion for IGD, but the withdrawal and relapse criteria largely capture it [54, 55], whereas conflict and salience capture harm-reward imbalance and interference attributable to cognitive hijacking (see also [19]). In other words, compulsivity seems to be at the very core of behavioral addiction.

In summary, although compulsivity seems to be a slightly better predictor of severity of gambling problems than of video gaming-related problems, and factors other than compulsivity might play a stronger role in video gaming-related than in gambling harms, compulsive behavior presents strikingly similar features in the two domains. Still, this similarity does not necessarily imply the existence of a common etiological pathway to gambling and gaming-related problems. Specifically, Heather [6] distinguishes between strong and weak conceptualizations of compulsivity. In the first sense, “compulsion is seen as an example of automatic, involuntary behaviour following repeated learning experiences” (p. 32). In the second sense, compulsion is seen as “resulting from a failure to resist abnormally strong desires to engage in addictive behaviour” (p. 32). These two versions mostly parallel the two general mechanisms of habit formation and reward overvaluation mentioned earlier, but these mechanisms are not mutually exclusive and their relative effects on behavior can be indistinguishable when assessing using the customary psychometric tools. Indeed, both are present in the GRACC scale and, according to our analyses, they are psychometrically inseparable.

So far, and applying a basic principle of parsimony, if compulsivity in gambling and video gaming domains is almost indistinguishable, the simplest answer to the etiology question is assuming the commonality of mechanisms. Any proposal of differentiable mechanisms across behavioral domains must be accompanied by testable predictions. Our tentative hypothesis for future investigation is that compulsive video gaming results from excessive valuation of gaming activities, and this, in turn, from lack of competition from alternative activities with sufficient potential to satisfy personal needs and goals, whereas compulsive gambling is more directly motivated by conditioned, cue-driven states (e.g., craving). If this hypothesis is correct, compulsive video gaming could be attributed to basic principles of operant learning and behavioral economics (see, for example [56]), whereas compulsive gambling would require specific conditioning mechanisms to account for progressive acquisition of urges and disproportionate short-term expectancies [57]. To date, both the role of need frustration and activity overvaluation in video gaming disorder [58–60], and the centrality of acute, cue-triggered states in gambling [61–63] have been extensively reported, but a direct comparison of trait and state predictors of compulsivity in both samples is pending. Unfortunately, in the present study, the only predictors collected were declarative motives, with largely different scales for each activity. Still, the ordering of correlations between motives and compulsivity seem to diverge to some degree, with coping/affect regulation motives playing a stronger role in compulsive gambling than in compulsive gaming (see [64, 65]; for converging evidence).

Our main proposals for future research are, first, to explore the mechanisms of compulsivity –once it has been precisely operationalized– by directly searching, not only the similarities, but also the potential differences, using comparable instruments; and, second, to incorporate qualitative analysis to the exploration of subjective and phenomenological experiences of compulsivity that also likely to differ across domains [66, 67].

### Limitations, strengths, and conclusions

This study has succeeded in delineating an operationalization of compulsivity, and developing a scale consisting only of highly discriminative items. However, this self-reported measure probably lacks the potential to provide evidence –by itself– on the etiology of compulsivity. Further translational, experimental, and process-based research is necessary to disentangle explanatory mechanisms.

Although some differential correlations open paths for future research (e.g., compulsivity seemingly playing a more central role in functional deterioration and harm in the gambling domain than in the video gaming domain), significant differences in correlation sizes between the two samples are small, and may result from the use of slightly different instruments across domains. Further research pursuing these differences should extend the use of the same instruments for the same constructs in different samples. Actually, the GRACC scale developed here could be an important step ahead in overcoming the fragmentation problem in behavioral addiction measurement. If there are common mechanisms for different putative behavioral addictions, research will certainly benefit from the existence of measures of those mechanisms that are applicable to different activities (instead of slightly different, non-comparable measures for each of them).

Actually, our scale is not the only available measure of compulsivity that can be applied across behavioral domains. On the one hand, the BATCAP [68] questions individuals about the following domains: alcohol use, gambling, compulsive eating, contamination compulsions, checking compulsions, just right and ordering compulsions, and compulsive Internet use. If the individual reports any of these behaviors in the last 30 days, they are asked to answer six further questions about time lost, distress, loss of control, functional impact, anxiety if prevented from doing the behavior, and strongest urge. This scale was developed following a theory-driven method, and was intended to detect transdiagnostic commonalities between addictive disorders and obsessive-compulsive disorder. There is some evidence, however, that compulsivity in the obsessive-compulsive spectrum and addictive disorders present important differential features, and does not necessarily constitute a single construct [69]. This evidence is consistent with our finding that items about inflexible rules and rituals –normally considered highly representative of obsessive-compulsive behavior– are the ones with lowest correlations with the common compulsivity factor in our analyses.

The GRACC has been however developed in a mostly data-driven manner, and is applicable to any putatively addictive activity (minimally rewording it to refer to the activity of interest). This data-driven method allowed us to start with a very large pool of items and then select the ones that best reflect the underlying construct. It is indeed reassuring that BATCAP items and Muela et al.’s operationalization largely overlap, but the GRACC goes a step further in showing that not all of those operationalizations are equally central for the conceptualization of compulsivity. Other compulsivity scales available in the literature, as the Cambridge-Chicago Compulsivity Trait Scale [70] (for a review see [71]), are not directly comparable to ours, as they measure compulsivity as a trait, that is, as a general proneness or vulnerability to develop compulsive behaviors. So, they cannot be used to determine whether or not specific activities have become compulsive. Our aim here is not to assess individuals’ traits, but a feature of a specific activity as currently presented by an individual.

Our data-driven approach was intentional. Indeed, this approach has shown that, although the six operationalizations identified by Muela et al. (Table 1) were conceptually different, in the end, all of them are too tightly correlated to be considered psychometrically separable dimensions. Although this is still highly speculative, urges and salience are probably two sides of the same coin, one reflecting motivational and affective aspects of craving (either appetitive or aversive), and the other reflecting the cognitive elaboration of desire. When these are strong enough, they end up causing the subjective feeling that the problematic behavior has escaped voluntary control, despite its harms having overridden its benefits. In other words, urgency and over-salience are possibly the core of compulsivity, and disutility is a sign that they are strong enough to override one’s goals. These behaviors are also likely to be perceived as habitual, stereotyped, or excessive, but these features are probably close correlates rather than key ingredients of compulsivity.

The multistep procedure followed in the development of the GRACC is probably the most detailed and systematic one in the fields of compulsivity and behavioral addictions to date. The result is a measure with outstanding psychometric properties, in terms of both reliability and convergent validity. Importantly, and despite its high correlations with severity measures, it goes beyond them in terms of intensionality (it does not depend on an extensional set of features). Specificity, however, does not come at the cost of predictive power, as compulsivity by itself accounts for as much variability in quality of life and negative affect measures as severity. This counts as strong evidence, by itself, that some symptoms are accessory to define a behavior as compulsive and eventually problematic.

## Data Availability

Supplementary materials and datasets generated and/or analysed during the current study are available in the Open Science Framework (OSF) repository as https://osf.io/xdfmw/?view_only=9831ce7702c34347ac67b45719ddf643.

## Abbreviations

BATCAP: Brief Assessment Tool for Compulsivity Associated Problems
bGMI: Brief Gambling Motives Inventory
CFI: Comparative Fix Index
DSM: Diagnostic and Statistical Manual of Mental Disorders
ESEM: Exploratory Structural Equation Model
FPS: First Person Shooters
GD: Gambling Disorder
GRACC: Granada Assessment for Cross-Domain Compulsivity
GRACC18: 18-item Granada Assessment for Cross-Domain Compulsivity
GRACC90: 90-item Granada Assessment for Cross-Domain Compulsivity
IGD: Internet Gaming Disorder
MMORPG: Massively Multiplayer Online Role-playing Game
MOBA: Multiplayer Online Battle Arena
PANAS: Positive and Negative Affect Scale
RMSEA: Root Mean Squared Error of Approximation
SMSR: Standardized Root Mean Square Residual
TECVASP: Quality of Life in Individuals Addicted to Psychoactive Substances Test
TLI: Tucker-Lewis Index
VMQ: Video-gaming Motives Questionnaire
WLSMV: Weighted Least Square Mean and Variance adjusted
